# Myristic Acid Remodels Sphingolipid Metabolism via Dual Pathways: Canonical d18-Sphingolipid Regulation and Non-Canonical d16-Sphingolipid Synthesis

**DOI:** 10.3390/nu17172881

**Published:** 2025-09-05

**Authors:** Yunfei You, Qinghe Zeng, Zhenying Hu, Yu Chen, Mengmin Zhan, Yanlu Wang, Jingjing Duan

**Affiliations:** Sphingolipid Metabolism and Aging, Human Aging Research Institute (HARI) and School of Life Science, Nanchang University, and Jiangxi Key Laboratory of Aging and Disease, Nanchang 330031, Chinawhozing@ncu.edu.cn (Z.H.); wangyanlu@ncu.edu.cn (Y.W.)

**Keywords:** myristic acid, sphingolipid metabolism, ceramides, d16-sphingolipids

## Abstract

**Background**: Myristic acid (MA), a 14-carbon saturated fatty acid, serves as a precursor for the synthesis of non-canonical d16-sphingoid bases via its activated form, C14:0-CoA. However, its broader regulatory role in sphingolipid (SL) metabolism remains poorly defined. **Methods**: Using HepG2 cells treated with 50 μM MA, we found that sphingolipidomic analysis revealed reprogrammed sphingolipid metabolism. **Results**: In the canonical d18-SL pathway, MA directs its activated product C14:0-CoA into ceramide *N*-acyl chains and downstream metabolites—especially d18:1-C14:0 hexosylceramide. Concurrently, in the non-canonical d16-SL pathway, MA promotes d16-SL synthesis, especially d16:1-ceramides (Cer), d16:1-hexosylceramides (HexCer), and d16:1-C14:0 lactosylceramide. MA treatment further induced a coordinated shift in cellular sphingolipid pools, characterized by a significant increase in total ceramide levels (encompassing both d16- and d18-species) alongside concurrent reductions in total sphingomyelin (SM) contents. At the gene transcriptional level, MA significantly suppressed *SPTLC2* mRNA expression while markedly upregulating *SMPD2* and *SMPD3* mRNA levels. **Conclusions**: Collectively, these findings position MA as a potent regulator of sphingolipid homeostasis, orchestrating dual pathway modulation: disrupting canonical d18-SL equilibrium through the selective enrichment of *N*-acyl C14:0-containing SLs, and activating non-canonical d16-SL synthesis. This dual pathway regulation reveals that dietary saturated fatty acids exploit sphingolipid subnetworks to regulate lipid metabolism. The interplay between dietary fatty acids and sphingolipid metabolism still requires deeper exploration. Our findings offer preliminary insights into their roles in regulating both normal and disease-associated lipid metabolism, setting the stage for subsequent mechanistic investigations.

## 1. Introduction

Myristic acid (MA), a 14-carbon saturated fatty acid, is predominantly sourced from milk fat, coconut oil, and palm kernel oil, accounts for approximately 0.07–0.44% (up to 2.0% in some regions) of total dietary energy [1,2]. Following intestinal absorption, it is catalyzed by acyl-CoA synthetase to form the key metabolic intermediate myristoyl-CoA (C14:0-CoA) [3]. This activated MA not only provides energy via *β*-oxidation and modulates plasma, liver, and adipose tissue fatty acid profiles [3,4], but also serves as a crucial substrate for several important biochemical processes, including protein *N*-myristoylation and the biosynthesis of triacylglycerols, phospholipids, and sphingolipids [5,6].

MA may exert dualistic effects on health, simultaneously exhibiting both protective and detrimental impacts depending on physiological context. Moderate amounts of MA not only mitigate age-related intervertebral disk degeneration in naturally aging mice by suppressing endplate chondral remodeling and preventing vertebral osteoporosis (10 and 50 μM MA) [7], but also enhance triglyceride and fatty acid deposition in broiler chicken pectoralis muscle to improve muscle quality (0.04% MA in diet) [8]. On the other hand, excessive MA disrupts systemic lipid homeostasis by elevating total cholesterol, LDL-C, and HDL-C levels while exacerbating ceramide-dependent lipotoxicity [9,10], and contributes to pathological tissue remodeling through C14-ceramides (Cer)-mediated myocardial hypertrophy and intestinal stress, concomitantly impairing cell membrane function and signal transduction [3,5,11,12,13]. These dichotomous effects arise from MA’s involvement in activating TLR4/NF-κB signaling to promote inflammation [14,15], while also facilitating oncogenic pathways through protein *N*-myristoylation [16]. Notably, it has been found that MA’s sphingolipid metabolic remodeling, characterized by C14-Cer generation, simultaneously influences cell membrane dynamics and intercellular communication, underscoring its role in sphingolipid (SL) metabolic regulation, which plays a crucial part in cellular function.

**Figure 1:**
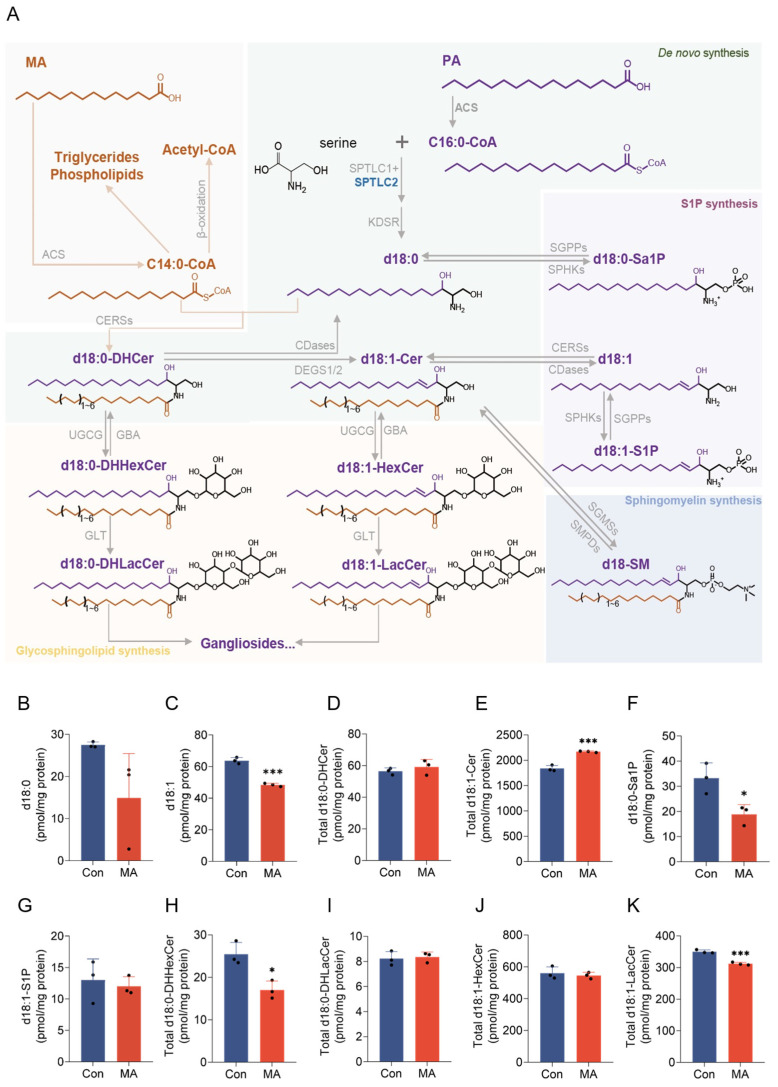
Myristic acid (MA) induces reprogramming of canonical d18-sphingolipid metabolism. (**A**) Schematic representation of the involvement of MA in canonical d18-sphingolipid (SL) metabolic pathway. (**B**–**K**) Quantitative analysis of MA-induced alterations in d18-sphingolipid species (units: pmol/mg protein). (**B**,**C**) Sphingoid base measurements showed reduced d18:0 (**B**) and d18:1 (**C**) levels. (**D**,**E**) Ceramide analysis demonstrated unchanged d18:0-dihydroceramides (DHCer, (**D**)) and increased d18:1-ceramides (Cer, (**E**)). (**F**,**G**) Sphingoid base-1-phosphate (S1P) quantification revealed reduced d18:0-sphinganine-1-phosphate (Sa1P, (**F**)) and d18:1-S1P (**G**). (**H**–**K**) Glycosphingolipid (GSL) measurements showed decreased d18:0-dihydrohexosylceramides (DHHexCer, sum of dihydro glucosyl- and dihydrogalactosyl-ceramides, (**H**)), and d18:1-lactosylceramides (LacCer, (**K**)), while d18:0-dihydrolactosylceramides (DHLacCer, (**I**)) and d18:1-hexosylceramides (HexCer, sum of glucosyl- and galactosyl-ceramides, (**J**)) showed no significant change. Data are from three independent biological replicates as the black dots indicated. The *p* values are compared with the control group, and analyzed using a two-tailed Student’s *t*-test, with * *p* < 0.05, and *** *p* < 0.001.

Ceramides sit at the metabolic crossroads of sphingolipid biosynthesis, serving as the central node where three primary synthetic routes converge: *de novo* synthesis from sphingoid bases, hydrolysis of complex sphingolipids including glycosphingolipids (GSLs) and sphingomyelins (SMs), and the salvage pathway that recycles sphingoid base-1-phosphate (S1P) [17]. The *de novo* synthesis pathway is initiated by the serine palmitoyltransferase (SPT) [17]. The SPT complex is composed of SPTLC1 and SPTLC2 subunits that preferentially utilizes palmitoyl-CoA (C16:0-CoA) and serine as substrates to produce an 18-carbon backbone sphinganine, d18:0 [17]. SPT can also utilize alanine in place of serine to generate 18-carbon backbone 1-deoxysphinganine, m18:0 [18]. The sphinganine intermediate is subsequently *N*-acylated by ceramide synthases (CERSs) to form dihydroceramides (DHCer), which are then desaturated by desaturase enzyme (DEGS) to yield canonical d18-Cer (Figure 1A) [17]. The C14:0-CoA derived from MA can be utilized by SPTLC1 and SPTLC3 composed SPT complex as a substitute for C16:0-CoA, catalyzing the synthesis of non-canonical d16-sphingoid bases characterized by a 16-carbon sphingosine backbone [19]. In this way, MA serves as a key regulatory molecule in maintaining sphingolipid homeostasis.

While MA has been shown to participate in the synthesis of non-canonical d16-sphingoid bases and d16:1-Cer, its regulatory role in canonical d18-SL metabolism, and whether MA affects the generation of downstream d16:1-complex sphingolipids, remain poorly understood. To address this knowledge gap, we employed HepG2 cells as an in vitro model of hepatic fatty acid metabolism, combined with sphingolipidomic analysis to investigate the effects of MA on both canonical d18-SLs and non-canonical d16-SLs. This approach aims to provide novel insights into the impact of MA on sphingolipid homeostasis.

## 2. Materials and Methods

### 2.1. Reagents

The myristic acid (MA, Sigma, St. Louis, MO, USA, #M3128, purity ≥ 98%, BHT-free) was sourced in powder form, dissolved in ethanol to reach a 50 mM concentration for stock solutions, stored at −20 °C, and used within a week. To maintain analytical precision, fresh working solutions were made each day. The internal standard (Sphingolipid Mix II, Avanti Polar Lipids, Alabaster, AL, USA, #LM-6005, containing d17:0, d17:1, d17:0-sphinganine-1-phosphate (Sa1P) or d17:1-sphingosine-1-phosphate (S1P), C12-ceramide (Cer), C12-sphingomyelin (SM), C12-glucosylceramide (GluCer), C12-lactosylceramide (LacCer, D-lactosyl-ceramide) was confirmed to have a purity exceeding 95% and to be within 10% of the designated concentration (25 μM). For lipid extraction and LC-MS/MS analysis, HPLC-grade or higher purity solvents and chemicals were used, comprising methanol (Sigma, USA, #34860), isopropanol (Merck, Darmstadt, Germany, #1.01040), and formic acid (Macklin, Shanghai, China, #809712). Additional reagents from Sigma (USA) were chloroform (#39918; used for phase separation), dichloromethane (#34856; used for lipid layer separation), acetonitrile (#34851; low UV absorbance for MS compatibility), ammonium formate (#70221; 5 mM stock for buffer preparation), and ammonium acetate (#73594; applied for pH stabilization of samples).

### 2.2. Preparation and Treatment of Cell Cultures

HepG2 cells (Kunming, China, #KCB200507YJ) were routinely maintained in a complete growth medium consisting of the following: Dulbecco’s modified Eagle medium (DMEM, high glucose) purchased from Biological Industries (Beit HaEmek, Israel, #10-013), fetal bovine serum (FBS, heat-inactivated) supplied by Clarke Bioscience (Shanghai, China, #FB25015) at a final concentration of 10%, and penicillin-streptomycin (PS) from Solarbio (Beijing, China, #P1400) at a 1% working concentration.

A density of 1.0 × 10^7^ HepG2 cells per dish was used to seed 10 cm culture dishes, and they were cultured for 12 h under standard conditions. Unless stated otherwise, cells were then exposed to 50 µM MA for 24 h. Test compounds were prepared from ethanol-based stock solutions, diluted to working concentrations, and precomplexed with 10% bovine serum albumin (BSA, Sigma, USA, #V900933) for 30 min at 55 °C. This mixture was added to complete growth medium, immediately before cell treatment. The molar ratio of MA to BSA was 33.2:1 during the conjugation step. The final BSA concentration in all treatment and control media was 0.005%. The final ethanol concentration was 0.5 µL/mL of medium (0.05% *v*/*v*). Controls were exposed to ethanol (vehicle) alone at the same volume, conjugated with BSA using the same protocol. To exclude potential confounding effects of cytotoxicity, cell viability was assessed using an MTS assay (Promega, Madison, WI, USA, #G3580) after all treatments.

### 2.3. Lipid Extraction

Cells were collected at 80–90% confluence and then mechanically detached using a cell scraper, following two rinses with ice-cold phosphate-buffered saline (PBS). Cell pellets were gently resuspended in 150 μL of Milli-Q water to obtain a homogeneous suspension. Lipid extraction was conducted utilizing a modified version of the methodology out-lined in our prior research [20,21]. Single-phase and two-phase extractions were performed on the target sphingolipids. After adding the extraction solvent, 10 μL of internal standard was introduced as the internal control. The lipid extraction protocol involved overnight static incubation at 48 °C, followed by saponification, neutralization, sequential solvent extraction (primary and twice re-extraction). Detailed procedures are provided in Supplementary Table S1. The combined extracted phases were dried under a nitrogen stream. Prior to ultra-high performance liquid chromatography-tandem mass spectrometry (UPLC-MS/MS), the samples were reconstituted in 250 μL extract liquid, centrifuged at 12,000 rpm for 10 min, and the resulting supernatants were collected for sphingolipid analysis. Following centrifugation, each supernatant was applied to sphingolipid analysis. Data on the recovery of samples calculated against the internal standard and intra/inter-day coefficients of variation (intra/inter-day CV) are provided in Supplementary Table S2; based on the corrected values, the extraction efficiency of this method is over 80%.

### 2.4. LC-MS/MS Profiling

Sphingolipid analysis was conducted with an ultra-high performance liquid chromatography (UPLC, Shimadzu, Kyoto, Japan) system coupled to a Triple Quad™ 5500+ QTRAP™ MS (AB Sciex, Concord, ON, Canada), which included an LC-30AD binary pump, DGU-20A5 degasser, SIL-30AC autosampler, CTO-20AC column oven, and CBM-20A system controller [20,22]. The mass spectrometer (MS) was operated in electrospray ionization (ESI+) mode for multiple reaction monitoring (MRM) (Supplementary Table S3). The ion source parameters were configured as: ESI voltage, 4.5 kV; source temperature, 40 °C; ion source gas 1 (nebulizer), 60 psi; and curtain gas, 40 psi. Dwell duration was adjusted to 50 ms, and then to 5 ms for inter-channel delay. Analyst 1.7.3 software (Applied Biosystems, Waltham, MA, USA) was used for instrument control and data acquisition. The Pierce BCA protein assay Kit (Thermo Scientific, Waltham, MA, USA, #23225) was used to standardize the quantification of sphingolipids against the concentration of proteins. UPLC conditions for sphingolipid analysis are provided in Supplementary Table S4.

Quantification was performed using SCIX OS software (Version 3.0.0.3339, Applied Biosystems). Concentration of each target sphingolipid was calculated using the formula: picomoles of analyte = correction factor × (analyte peak area/internal standard peak area) × picomoles of internal standard added. Sphingolipids with *N*-acyl chain lengths (C12, C16:0, C18:1, C20:0, C24:0, and C24:1) were quantified by comparison with authentic standards and their corresponding internal standards: C12 Cer, SM, GluCer and LacCer. Correction factors for other *N*-acyl chain length species (C18:0, C20:1, C22:0, and C26:0) were derived from standards based on the observed relationship between chain length and peak area [23]. The internal standard was the C12-Cer, which was used to quantify Cer. Similarly, internal standards were applied to SMs, hexosylceramides (HexCer, which represent the total of isomers glucosyl-and galactosyl-ceramides) and LacCer using the following respective standards: C12-GluCer (for HexCer and SMs), and C12-LacCer (for LacCer). Sphingoid bases and sphingoid base-1-phosphate were quantified by utilizing d17:0, d17:1, d17:0-Sa1P or d17:1-S1P as internal standards. The developed LC-MS/MS method was validated for linearity, limit of detection (LOD), and lower limit of quantification (LLOQ); inter- and intra-day precision (%CV) were below 15% (Supplementary Table S2), and all analytes demonstrated good stability under all tested conditions.

### 2.5. Real-Time PCR with Quantitative Analysis

Total RNA from MA-treated and untreated cells was extracted using RNAiso Plus from TaKaRa (Kusatsu, Japan, #9108), reverse-transcribed with the PrimeScript RT Kit (#RR047A) from the same manufacturer, and analyzed by quantitative real-time PCR (qPCR) using Mei5 Biotechnology’s 2× M5 HiPer SuperMix (Beijing, China, #MF013-01) with primers from Supplementary Table S5. The amplification efficiency of all primers was maintained between 90% and 110%. Relative gene expression was determined by the 2^−ΔΔCt^ method with *GAPDH* as the control.

### 2.6. Statistical Analysis

Data are presented as the mean ± standard deviation (mean ± SD). An un-paired two-tailed Student’s *t*-test was used to analyze the differences between the MA-treated and untreated groups, with homogeneity of variances confirmed by *F*-test and Welch’s correction applied for unequal variances; the thresholds for statistical significance were * *p* < 0.05, ** *p* < 0.01, and *** *p* < 0.001. GraphPad Prism 10.0 from La Jolla (CA, USA) and the R pheatmap package were employed to generate figures. Further details about the analysis parameters and calculated content of each sphingolipid, the *p* values, Cohen’s d, and confidence intervals (CIs) can be requested from the authors.

## 3. Results

### 3.1. Myristic Acid Reprograms Canonical d18-Sphingolipid Metabolism

Myristic acid (MA) is primarily activated to C14:0-CoA by acyl-CoA synthetase (ACS) and then metabolized via *β*-oxidation for energy production or esterified into triglycerides/phospholipids (Figure 1A) [3,24]. A portion of C14:0-CoA undergoes elongation to C16:0 by elongases of very long chain fatty acids (ELOVL) [25], while the remainder directly incorporates into ceramides (Cer) or regulates *de novo* sphingolipid synthesis for canonical d18-sphingolipid productions (Figure 1A). To elucidate the impact of MA on canonical d18-sphingolipid profiles, we performed targeted quantification analysis of canonical sphingoid bases including d18:0 and d18:1, Cer including d18:0-dihydroceramides (DHCer) and d18:1-Cer, phosphorylated sphingoid bases d18:0-sphinganine-1-phosphate (Sa1P) and d18:1-sphingosine-1-phosphate (S1P), and glycosphingolipids comprising dihydrohexosylceramides (DHHexCer), hexosylceramides (HexCer), dihydrolactosylceramides (DHLacCer), and lactosylceramides (LacCer).

We applied a 50 μM treatment concentration of MA for subsequent experiments [26], which was verified to exert no cytotoxic effects on cell viability (Figure S1). Quantitative analysis of d18-sphingolipids in MA-treated HepG2 cells revealed a complex remodeling of sphingolipid metabolism. While sphinganine d18:0, the product of ketosphinganine reductase (KDSR), exhibited a non-significant decreasing trend (Figure 1B), sphingosine d18:1 was significantly reduced (Figure 1C), indicating diminished *de novo* synthesis of canonical sphingolipids. The m18:0 and m18:1 sphingoid bases, generated through serine palmitoyltransferase (SPT)-catalyzed incorporation of alanine instead of serine as the substrate, were also significantly reduced (Figure S2A,B), whereas the level of m18:1-C18:0 ceramide was significantly increased (Figure S2L), with m18-Cer remaining unchanged (Figure S2G,H). In contrast, d18:0-DHCer remained unchanged (Figure 1D), but d18:1-Cer levels, which are primarily synthesized by ceramide synthases (CERSs), increased markedly (Figure 1E). This was accompanied by a significant reduction in d18:0-Sa1P (Figure 1F) and unchanged d18:1-S1P level (Figure 1G).

In glycosphingolipid synthesis, total d18:0-DHHexCer, the products of UDP-glucose ceramide glucosyltransferase (UGCG), was markedly decreased (Figure 1H), whereas d18:0-DHLacCer remained unaffected (Figure 1I). The levels of d18:1-HexCer, a substrate for glucosylceramidase (GBA), were stable (Figure 1J), but d18:1-LacCer, synthesis of which were catalyzed by lactosylceramide synthase (GLT), were significantly reduced (Figure 1K). Collectively, these findings demonstrate that MA reprograms d18-sphingolipid metabolism and preferentially drives d18:1-Cer synthesis while simultaneously inhibiting its conversion to GSLs.

### 3.2. Myristic Acid Suppresses SPTLC2 mRNA Expression and Directs C14:0-CoA into d18:1-Cer/HexCer

**Figure 2:**
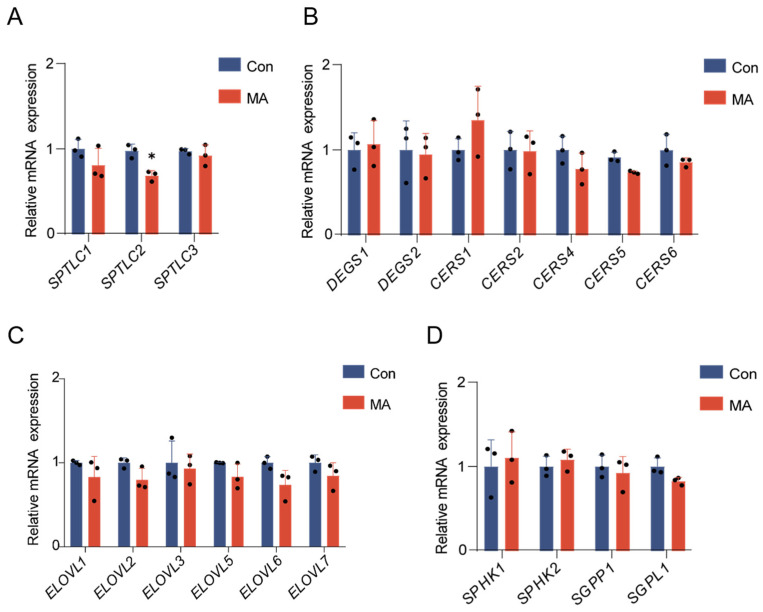
MA modulates sphingolipid-related gene expression profiles. Quantitative PCR analysis of the mRNA expression of serine palmitoyltransferase (SPT) subunit genes (*SPTLC1-3*) (**A**), genes for ceramide synthesis (*DEGS1-2*, *CERS1-6*) (**B**), fatty acid elongase genes (*ELOVL1-7*) (**C**) and S1P synthesis and cleavage genes (*SPHK1-2*, *SGPP1*, *SGPL1*) (**D**). The *p* values are compared to the control group without MA supplementary cells, analyzed using a two-tailed Student’s *t*-test from three independent biological experiments (black dots represent individual replicates), with * *p* < 0.05.

Given the observed alterations in sphingolipid composition, characterized by significant suppression of d18:1 and GSLs including d18:0-DHHexCer and d18:1-LacCer alongside marked elevation of d18:1-Cer, we next examined the mRNA expression levels of key synthetic enzymes involved in their metabolism. Quantitative PCR analysis revealed a downregulation of *SPTLC2* mRNA level among SPT, while with no significant changes in *SPTLC1* or *SPTLC3* mRNA expression (Figure 2A), suggesting that MA treatment reduced d18-sphingoid bases potentially involving transcriptional modulation of *SPTLC2*, whereas the elevated d16-sphingoid bases may not be directly linked to changes in *SPTLC3* transcription. Notably, no statistically significant differences were detected in the mRNA expression of *DEGS1-2*, *CERS1-6* or *ELOVL1-7* (Figure 2B,C), indicating that the increased d18:1-Cer levels are unlikely driven by transcriptional upregulation of ceramide synthases or *ELOVLs*. It is also noteworthy that d18:0-Sa1P was significantly reduced, whereas the mRNA expression levels of sphingosine kinases (*SPHK1-2*), S1P phosphatase (*SGPP1*), and S1P lyase (*SGPL1*) remained unchanged (Figure 2D).

**Figure 3:**
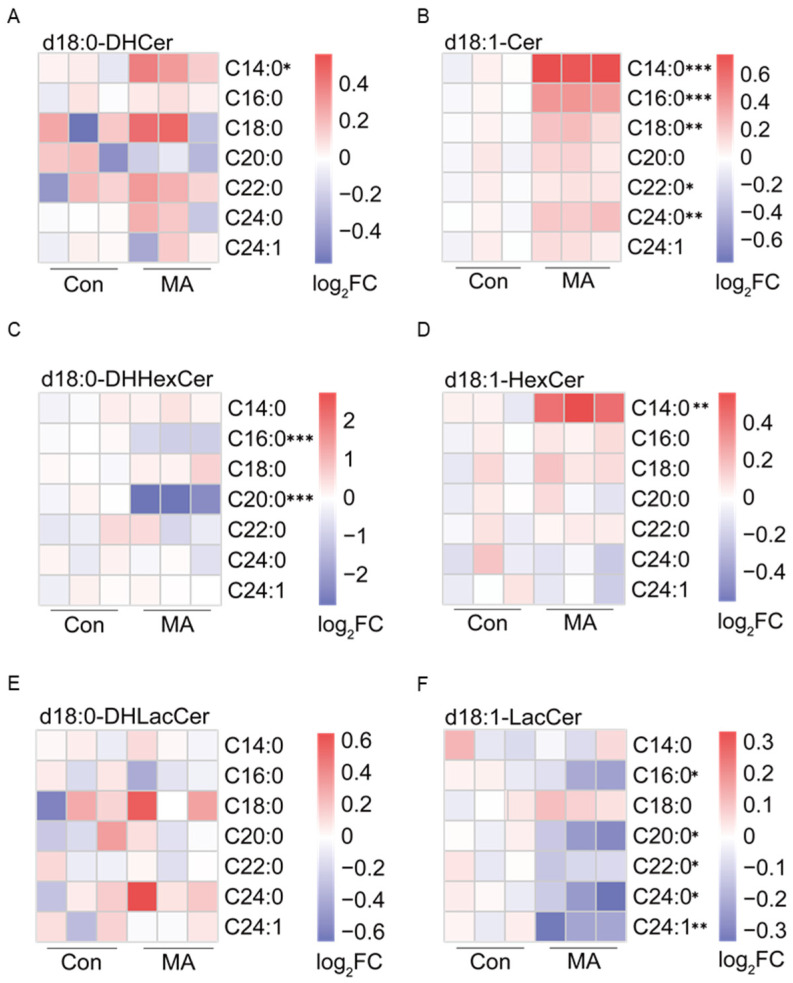
MA directs C14:0-CoA incorporation into d18:1-Cer and d18:1-HexCer. (**A**,**B**) Heatmap visualization of log_2_-fold changes in *N*-acyl chain length distribution for (**A**) d18:0-DHCer and (**B**) d18:1-Cer, showed preferential incorporation of C14:0 into d18:1-Cer. (**C**,**D**) Quantitative analysis of the levels of (**C**) d18:0-DHHexCer and (**D**) d18:1-HexCer demonstrated a significant enrichment of *N*-acyl C14:0-containing d18:1-HexCer. (**E**,**F**) Quantification of (**E**) d18:0-DHLacCer and (**F**) d18:1-LacCer revealed divergent regulation between these glycosphingolipids species. Color scale: white represents the mean of all samples, with increasing red intensity indicating higher lipid levels and increasing blue intensity indicating lower levels. For each panel, the maximum positive (red) log_2_FC values and their corresponding fold changes are: 0.4 (~1.3-fold); 0.6 (~1.5-fold); 2.0 (4.0-fold); 0.3 (~1.2-fold). A log_2_FC value of 0 indicates no change from the mean, with positive and negative values representing upward and downward regulation, respectively. The *p* values are compared to the control group without MA supplementary cells, analyzed using a two-tailed Student’s *t*-test from three separate biological experiments, with * *p* < 0.05, ** *p* < 0.01, and *** *p* < 0.001.

To further elucidate the compositional changes in canonical d18-sphingolipids, we analyzed the *N*-acyl side chain profiles, which revealed distinct remodeling patterns. In d18:0-DHCer, the *N*-acyl C14:0 showed the most significant increase while C20:0 exhibited a tendency toward downregulation (Figure 3A). The elevation of total d18:1-Cer level was driven not only by the *N*-acyl C14:0 but also by significant increases in C16:0, C18:0, C22:0 and C24:0 side chains (Figure 3B), confirming that MA-derived C14:0-CoA is incorporated into d18-Cer, with potential contributions from elongation of the C14:0 moiety to longer *N*-acyl chains. While d18:0-DHHexCer exhibited significant downregulation of C16:0 and C20:0 side chains (Figure 3C), d18:1-HexCer showed a marked increase in the *N*-acyl C14:0 content accompanied by a clear trend toward elevated levels of C16:0, C18:0, C20:0, and C22:0 (Figure 3D), alongside decreases in C24:0 and C24:1. These changes collectively confirm that MA-derived C14:0-CoA is actively incorporated into d18:1-HexCer, with potential contributions from *N*-acyl C14:0 elongation to longer-chain variants.

For downstream metabolites, d18:0-DHLacCer displayed no significant alterations in *N*-acyl chain composition (Figure 3E), whereas total d18:1-LacCer levels exhibited a marked decrease, particularly in C16:0, C20:0, C22:0, C24:0, and C24:1 (Figure 3F), with *N*-acyl C14:0-containing d18-LacCer remaining unchanged (Figure 3E,F). This selective exclusion of *N*-acyl C14:0 from d18-LacCer suggests that MA-derived C14:0-CoA was not efficiently channeled into lactosylceramide synthesis. These findings demonstrate that MA exerts subtype-specific effects on d18-sphingolipid metabolism, preferentially incorporating *N*-acyl C14:0 into d18:1-Cer and d18:1-HexCer, while blocking its further conversion to d18:1-LacCer.

### 3.3. Myristic Acid Drives the Non-Canonical d16-Sphingolipid Biosynthesis

**Figure 4:**
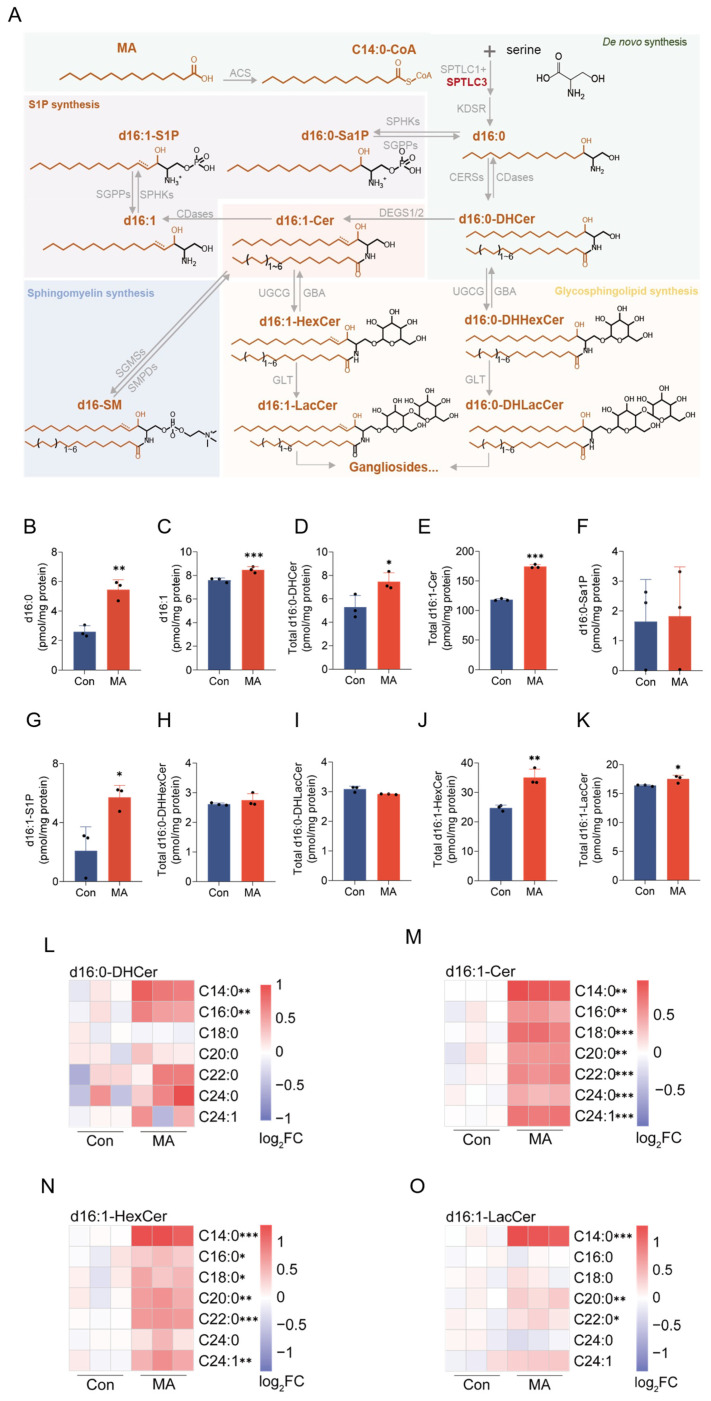
MA drives the non-canonical d16-sphingolipid biosynthetic pathway. (**A**) Schematic representation of the d16-sphingolipid synthesis pathway. (**B**,**C**) Quantification of free sphingoid bases showed elevated levels of (**B**) d16:0 and (**C**) d16:1 (units: pmol/mg protein). (**D**,**E**) Ceramide analysis revealed increased (**D**) d16:0-DHCer and (**E**) d16:1-Cer levels (units: pmol/mg protein). (**F**,**G**) S1P quantification demonstrated (**F**) non-affected d16:0-Sa1P and (**G**) enhanced d16:1-S1P production (units: pmol/mg protein). (**H**–**K**) GSL measurements revealed increases in d16:0-DHHexCer (**H**) and d16:0-DHLacCer (**I**), while d16:1-HexCer (**J**) and d16:1-LacCer (**K**) were also significantly elevated (units: pmol/mg protein). (**L**–**O**) Heatmap analyses of *N*-acyl chain length distributions (C14~C24) for (**L**) d16:0-DHCer, (**M**) d16:1-Cer, (**N**) d16:1-HexCer, and (**O**) d16:1-LacCer, with log_2_-fold changes indicating MA-induced remodeling. Color scale: white color denotes the mean value across all samples, where progressive reddening reflects higher lipid levels and progressive bluing reflects lower levels. For each panel, the maximum positive (red) log_2_FC values and their corresponding fold changes are: 1.0 (2.0-fold), 0.5 (~1.4-fold). A log_2_FC value of 0 indicates no change from the mean, with positive and negative values representing upward and downward regulation, respectively. The *p* values are compared to the control group without MA supplementary cells, analyzed using a two-tailed Student’s *t*-test from three separate biological experiments, with * *p* < 0.05; ** *p* < 0.01; and *** *p* < 0.001.

MA-derived C14:0-CoA serves as a substrate for SPTLC3, the catalytic subunit of the SPT complex, driving the synthesis of d16-sphingoid bases [19]. To characterize the resulting d16-sphingolipid species, we performed targeted quantitative sphingolipidomics (Figure 4A). MA treatment significantly elevated d16-sphingoid bases d16:0 (Figure 4B) and d16:1 (Figure 4C). Notably, d16:0 exhibited a greater fold-change than d16:1, indicating the robust activation of *de novo* sphingolipid synthesis despite unchanged *SPTLC3* mRNA levels.

The elevated d16-sphingoid bases were efficiently channeled into ceramide synthesis, as evidenced by increased d16:0-DHCer (Figure 4D) and d16:1-Cer (Figure 4E). Alternatively, sphingoid bases could be phosphorylated by sphingosine kinases (SPHKs) [17]. While d16:0-Sa1P levels were not affected (Figure 4F), d16:1-S1P showed a significant increase (Figure 4G). In glycosphingolipid biosynthesis, ceramides serve as substrates for sequential glycosylation to form HexCer and LacCer [17]. Analysis of d16-glycosphingolipids revealed unchanged d16:0-DHHexCer (Figure 4H) and d16:0-DHLacCer (Figure 4I), whereas d16:1-HexCer (Figure 4J) and d16:1-LacCer (Figure 4K) were significantly upregulated. Collectively, these results demonstrate that MA elevates free d16-sphingoid bases, d16:1-S1P, Cer and GSLs, while the biosynthetic pathways for non-canonical d16-sphingolipids remain functionally intact. The preferential elevation of d16:1-species suggests MA may preferentially channel d16:0-sphingoid base toward specific downstream products rather than uniformly activating the entire pathway.

To assess compositional changes in DHCer, Cer, HexCer, and LacCer across *N*-acyl chain lengths, we quantified d16-sphingolipid species using mass spectrometry and visualized alterations through fold-change calculations. Quantitative analysis of d16-sphingolipids across acyl chain lengths revealed that MA treatment consistently increased d16:0-DHCer levels (except C18:0), with pronounced elevation at *N*-acyl C14:0 and C16:0 (Figure 4L). It also enhanced d16:1-Cer (Figure 4M) and d16:1-HexCer (Figure 4N) across *N*-acyl C14~C24 chains. Conversely, d16:1-LacCer increased only at *N*-acyl C14:0, C20:0, and C22:0, with no significant differences observed at other chain lengths (Figure 4O). Notably, among d16:0- and d16:1-(DH)Cer, (DH)HexCer, and (DH)LacCer, the molecular subtypes acylated with C14:0 all showed the most significant elevation (Figure 4L–O and Figure S3). These findings demonstrate MA-derived C14:0-CoA is specifically incorporated into Cer and efficiently metabolized to downstream HexCer and LacCer. In summary, MA-derived C14:0-CoA integrates into d16-sphingolipids, driving efficient metabolism through Cer to S1P and GSL pathways, with *N*-acyl C14:0-acylated species exhibiting the most pronounced increases at all metabolic nodes. These results indicate that MA specifically activates the d16-sphingolipid pathway.

In addition, among non-canonical sphingolipids, the SPTLC3 can also utilize alternative fatty acids to synthesize d19- and d20-series sphingolipids [26,27]. Our analysis revealed that while d19:1-sphingoid base level was significantly reduced, other d19- and d20-series free bases and corresponding ceramides remained unchanged (Figure S2C–N).

### 3.4. Myristic Acid Increases Total Cer but Inhibits Total Glycosphingolipids and Sphingomyelins

**Figure 5:**
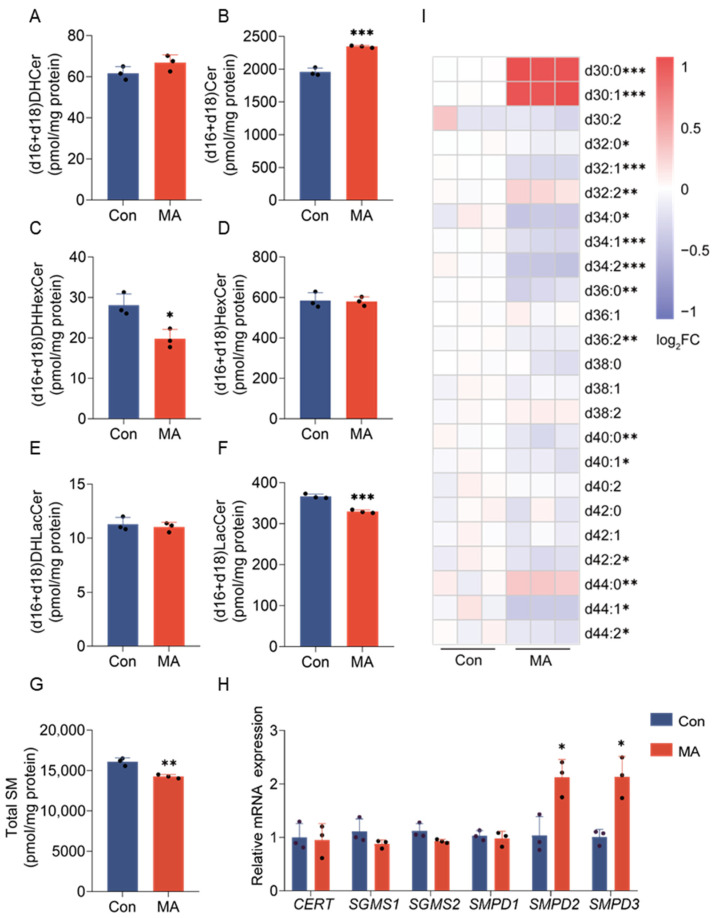
MA induces sphingolipid metabolic remodeling via mRNA-level upregulation of *SMPD2* and *SMPD3*, potentially driving sphingomyelin (SM) degradation and ceramide elevation. (**A**–**G**) Quantitative analysis of total lipid contents for d18- and d16-series sphingolipids (units: pmol/mg protein): d18- and d16- total (**A**) DHCer, (**B**) Cer, (**C**) DHHexCer, (**D**) HexCer, (**E**) DHLacCer, (**F**) LacCer, and (**G**) total SM. (**H**) The mRNA expression of genes involved in ceramide transfer protein (CERT) and SM metabolism (SGMS1-2, SMPD1-3) in HepG2 cells after 24 h MA treatment. (**I**) Heatmap showed log_2_-fold changes in SM acyl chain length distribution (d30~d44; e.g., d30 likely denotes a d16-sphingoid base with a C14 *N*-acyl chain) in MA-treated HepG2 cells. Color scale: white denotes the sample mean, with intensifying red indicating elevated lipid levels and deepening blue signifying reduced levels. The scale is calibrated such that the maximum positive (red) log_2_FC values correspond to 1.0 (2.0-fold increase), and 0.5 (~1.4-fold increase), respectively. A log_2_FC value of 0 indicates no change from the mean, with positive and negative values representing upward and downward regulation, respectively. The *p* values are compared to the control group without MA supplementary cells, analyzed using a two-tailed Student’s *t*-test from three separate biological experiments (black dots represent individual replicates), with * *p* < 0.05; ** *p* < 0.01, and *** *p* < 0.001.

Sphingolipid function is determined by both their molecular composition and quantitative abundance. Building on our analysis of individual d18- and d16-sphingolipid species, we further quantified the total pool of these lipids to evaluate their comprehensive metabolic impact. Metabolite profiling revealed that while total d18- and d16-DHCer levels remained unchanged (Figure 5A), total ceramide contents were significantly elevated (Figure 5B). Regarding GSLs, DHHexCer levels decreased markedly (Figure 5C), whereas downstream HexCer remained stable (Figure 5D). Similarly, DHLacCer showed no change (Figure 5E), but LacCer contents were significantly reduced (Figure 5F). In addition to GSLs, MA treatment significantly reduced total sphingomyelin (SM) contents (Figure 5G). However, this overall decrease exhibited pronounced molecular subtype specificity: while most SM subtypes—including d32:0, d32:1, d34:0, d34:1, d34:2, d36:0, d36:2, d40:0, d40:1, d42:2, d44:1, and d44:2—were markedly downregulated (Figure 5I), specific subtypes (d30:0-SM, d30:1-SM, d32:2-SM, and d44:0-SM) showed significant increases. Notably, the proportion of d30-SM within total SM rose from 3.83 to 4.05% to 5.40–5.58% post-treatment. The d30:0-SM and d30:1-SM likely correspond to d16:0-C14:0 and d16:1-C14:0 sphingomyelins, respectively. This dichotomy indicates that, aside from C14:0-containing d16-Cer serving as a precursor for specific elevated SM species, the majority of SM subtypes were reduced by MA treatment.

SM is synthesized via the choline headgroup transfer reaction catalyzed by sphingomyelin synthase, where ceramide is transported to the Golgi apparatus by CERT, and is primarily degraded by sphingomyelinases SMPD1-3 [17]. To better understand the cause of the substantial decrease in total SM contents, we analyzed the expression levels of key genes regulating its synthesis (*CERT*, *SGMS1-2*) and degradation (*SMPD1-3*). The expression of SM phosphodiesterase genes *SMPD2* and *SMPD3* was significantly upregulated (Figure 5H), suggesting that MA may promote SM degradation by enhancing the expression of these genes. While the mRNA expression of *CERT*, which encodes the ceramide transport protein, and SM synthase genes *SGMS1*-*2* was unaffected (Figure 5H).

## 4. Discussion

Our current study demonstrates that myristic acid (MA) exerts complex and selective effects on sphingolipid metabolism via multiple pathways: regulation of canonical d18-sphingolipids and stimulation of non-canonical d16-sphingolipid synthesis. Through its incorporation as C14:0-CoA, MA preferentially generates d18:1-ceramides (Cer), but suppresses d18-sphingolipid synthesis by downregulating *SPTLC2* mRNA expression, reducing free sphingoid bases. Simultaneously, MA drives non-canonical d16-sphingolipid production by enabling SPTLC3-dependent synthesis of d16-sphingoid bases, leading to elevated d16-Cer and d16:1-glycosphingolipids (GSLs) such as hexosylceramides (HexCer) and lactosylceramides (LacCer). Additionally, MA enhances sphingomyelin (SM) degradation, potentially via mechanisms involving the upregulation of *SMPD2* and *SMPD3* mRNA, selectively depletes major SM subtypes, and diminishes d18-GSLs including d18:0-dihydrohexosylceramides (DHHexCer) and d18:1-LacCer. Notably, the marked reduction in total sphingomyelin (SM) levels coupled with ceramide accumulation, alongside significant upregulation of *SMPD2* and *SMPD3* mRNA expression, suggests that MA-induced ceramide accumulation may arise from enhanced SM hydrolysis (i.e., SM⟶Cer flux). However, it is important to clarify that increased mRNA expression of *SMPD2* and *SMPD3* does not necessarily correlate with elevated enzyme protein levels or enhanced sphingomyelinase (SMase) activities. Future studies should investigate SMase regulation—for example, by applying specific inhibitors—to validate this mechanistic hypothesis. Furthermore, CERT (ceramide transporter) is a critical mediator of ceramide trafficking from the endoplasmic reticulum to the Golgi apparatus, where ceramide is converted to SM, thereby involving in the Cer⟶SM flux. Although MA treatment did not modulate *CERT* mRNA expression, the pronounced alteration in the Cer/SM balance warrants deeper investigation into CERT and sphingomyelin synthase’s regulatory role in this context. It should also be emphasized that this study was performed using a single concentration (50 μM MA) in HepG2 cells, without evaluating dose–response relationships or temporal dynamics. Future research should systematically explore these parameters to provide a more comprehensive mechanistic understanding. Collectively, these coordinated changes establish a distinctive metabolic signature characterized by elevated total Cer, reduced complex sphingolipids (DHHexCer, LacCer, SM), and demonstrate MA’s ability to differentially modulate lipid flux at multiple nodes, with potential implications for cellular homeostasis and signaling.

As key precursors for sphingolipid synthesis, the dietary fatty acids participate in the metabolic balance of sphingolipids, and this balance in turn affects physiological functions such as cellular signal transduction [28,29]. For canonical d18-sphingolipids, MA provides substrates for ceramide synthesis by increasing intracellular C14:0-CoA concentrations, leading to increases in d18:1-Cer (C14~C24). The SPTLC1/SPTLC2 complex catalyzes the *de novo* synthesis of canonical d18-sphingolipids (e.g., d18:0) from serine and palmitoyl-CoA, whereas the SPTLC1/SPTLC3 complex is responsible for producing canonical d16-sphingoid bases (e.g., d16:0) using myristoyl-CoA [26]. Notably, the significant reduction in *SPTLC2* mRNA expression, accompanied by a partial decline in *SPTLC1* mRNA level, along with decreased levels of d18:0 and d18:1, collectively indicates impaired *de novo* biosynthesis of d18-sphingolipids. This transcriptional disruption is more likely attributed to feedback inhibition triggered by the accumulation of total ceramides (Cer) [30], as the SPTLC3 mRNA level showed no significant change—suggesting that direct effects of MA treatment are less probable, and the regulation may be predominantly mediated by total Cer-induced negative feedback. Nevertheless, MA treatment reshapes the homeostatic balance between d18- and d16- sphingolipids, along with altering the overall sphingolipid profile. Given that our current data on serine palmitoyltransferase (SPT) subunits are limited to the mRNA level, highlighting a critical gap, future studies should employ Western blotting and SPT activity assays to determine whether MA regulates the protein level and function of SPT through translational or post-translational mechanisms, and ^13^C-labeled MA tracing experiments would enable the precise evaluation of MA’s metabolic flux into distinct d18- versus d16-sphingolipid pools, as well as its distribution into other lipid classes (e.g., triglycerides, phospholipids). Additional investigations such as co-treatment with palmitate are needed to elucidate how MA modulates the balance of sphingoid bases (e.g., d18, d16) and derived sphingolipids.

Current knowledge regarding the functional consequences of dietary MA uptake and the physiological significance of d16-sphingolipids remains limited. It is demonstrated that MA-derived C14:0-CoA is utilized by SPTLC3 to generate non-canonical d16-sphingoid bases and Cer [19]. Lone et al. demonstrated that only *SPTLC3*-overexpressed cells were capable of synthesizing canonical d16-sphingolipids upon MA supplementation in HEK293 [26], and our recent study further confirmed a significant increase in d16:0 in the livers of *Sptlc3*-overexpressed mice (Figure S4). To investigate the function of SPTLC3 in this process, future studies should incorporate *SPTLC3* knockdown models combined with MA treatment to evaluate d16-sphingolipid production. The current study reveals that d16-Cer preferentially form downstream d16:1-GSLs including HexCer and LacCer, as well as SMs, rather than d16:0-types. This emerging class of non-canonical d16-sphingolipids has garnered increasing attention due to their significant elevation in multiple pathologies, including type 2 diabetes, insulin resistance, non-alcoholic fatty liver disease, and cardiovascular disease [31,32,33,34,35,36,37]. Notably, Kovilakath et al. recently identified that d16 non-canonical sphingolipids, particularly d16-LacCer, were critical mediators of myocardial injury in ischemic cardiomyopathy, where they disrupt mitochondrial membrane integrity and complex I subunit composition [36]. In MA-treated hepatic cells, we observed a significant increase in d16:1-LacCer levels, while strikingly, total LacCer levels (comprising both d18 and d16 species) were reduced. This selective redistribution of LacCer subtypes warrants further investigation into its potential impact on mitochondrial function and diseases. Sustained elevation of d16:1-sphingosine-1-phosphate (S1P) in rat intestinal IEC-6 cells following MA treatment occurred without concomitant changes in d16:0-sphinganine-1-phosphate (Sa1P) levels [38], paralleling our hepatic cell findings. Given that d16:1-S1P serves as a critical signaling regulator of cell survival similar to d18-S1P [39,40], our findings indicate that MA may participate in S1P-GPCR axis-mediated cell fate decisions.

At the systemic level, MA treatment increased total ceramide levels (d16 + d18) while decreasing complex sphingolipids (GSLs and SMs). Elevation in Cer may contribute to pathological processes including apoptosis, inflammation, stress responses, and metabolic homeostasis disruption [41,42,43,44], and diminished GSL biosynthesis restores insulin signaling in adipocytes, enhances adipogenesis, and alleviates skin inflammation [45,46]. Collectively, our results demonstrate that MA remodels the molecular composition of sphingolipid subtypes. These findings may be particularly relevant for patients with cardiovascular or metabolic disorders requiring specialized dietary interventions. However, further validation through co-treatment with other fatty acids—especially palmitate—is warranted to compare the effects of different saturated fatty acids on sphingolipid metabolic flux and to elucidate MA’s systemic role in sphingolipid metabolism-related health issues.

## 5. Conclusions

Our current study demonstrates that myristic acid (MA) exerts a dual regulatory effect on sphingolipid metabolism. One facet of this effect is that, through acyl-CoA pool expansion, MA achieves a nuanced sphingolipid remodeling, characterized by the enhanced synthesis of C14-sphingolipids, preferential enrichment of diverse canonical d18:1-ceramides (Cer), and a concurrent reduction in d18-complex sphingolipids. Another facet is that MA activates the *de novo* synthesis of non-canonical d16-sphingolipids, including d16-sphingoid bases, d16-Cer, d16-hexosylceramides (HexCer), d16-lactosylceramides (LacCer) and some potential d16-sphingomyelins (SMs). Concomitantly, MA treatment was associated with transcriptional regulation in sphingolipid metabolism-related enzymes, featuring decreased *SPTLC2* mRNA levels, and increased *SMPD2* and *SMPD3* mRNA levels. Our findings offer primary but novel insights into how the uptake of the dietary fatty acid MA regulates sphingolipid metabolism, providing valuable perspectives for understanding and addressing human health issues related to lipid metabolism disorders.

## Data Availability

The original contributions presented in the study are included in the article/Supplementary Materials, further inquiries can be directed to the corresponding author due to privacy reasons.

## Supplementary Materials

The following supporting information can be downloaded at: https://www.mdpi.com/article/10.3390/nu17172881/s1, Figure S1. Cell viabilities of HepG2 cells treated with various concentration of myristic acid. The viability of HepG2 cells treated with myristic acid (MA) (0-500 μM, 24 h) was evaluated with the MTS assay, normalized to untreated controls. Statistical analysis via a two-tailed Student’s *t*-test indicated no significant differences (all *p* > 0.05, ns) compared to the control. Data are from three independent biological replicates (black dots indicate individual replicates). Figure S2. The effect of MA on non-canonical m18- 1-deoxysphingolipids and d19-, d20- sphingolipids. (A,B) Quantification of 1-deoxysphingoid bases showed reduced levels of (A) m18:0 and (B) m18:1 (units: pmol/mg protein). (C–F) Analysis of d19- and d20- sphingoid bases demonstrated a decrease in (D) d19:1, whereas (C) d19:0, (E) d20:0, and (F) d20:1 remained unchanged (units: pmol/mg protein). (G–J) Corresponding ceramide analysis showed that the levels of (G) m18:0-DHCer, (H) m18:1-Cer, (I) d19:1-Cer, and (J) d20:1-Cer were unaffected (units: pmol/mg protein). (K,L) Heatmaps of m18:0-DHCer (K) and m18:1-Cer (L) *N*-acyl chain distributions (C14~C24) as log_2_-fold changes. (M,N) Heatmaps displaying d19:1-Cer (M) and d20:1-Cer (N) chain length modifications (C16~C24) as log_2_-fold changes. For each panel, the maximum positive (red) log_2_FC values and their corresponding fold changes are: 0.3 (~1.2-fold), 0.6 (~1.5-fold), 1 (2.0-fold), 2 (4.0-fold). A log_2_FC value of 0 indicates no change from the mean, with positive and negative values representing upward and downward regulation, respectively. Color scale: all analyses were conducted in MA-treated HepG2 cells. Data are from three independent biological replicates (black dots indicate individual replicates). Significance was assessed by a two-tailed *t*-test (* *p* < 0.05, ** *p* < 0.01). Figure S3. The effect of MA on d16:0-dihydrohexosylceramides (DHHexCer), and d16:0-dihydrolactosylceramides (DHLacCer). Heatmaps displaying (A) d16:0-DHHexCer and (B) d16:0-DHLacCer with C16~C24 acyl chain length as log_2_-fold changes. Color scale: all analyses were conducted in MA-treated HepG2 cells. Statistical significance was determined by two-tailed Student’s *t*-test (n = 3 independent experiments), with * *p* < 0.05, and ** *p* < 0.01. Figure S4. Fold change in sphinganine d16:0 in WT and *Sptlc3*^OE^ mice at 6 months of age. Liver samples were collected from wild type (WT) and *Sptlc3* over-expressed C57BL/6J mice (SPF-grade, 6-month-old, males), with six mice per group (black dots indicate individual replicates). Statistical significance was determined by two-tailed Student’s *t*-test, with *** *p* < 0.001. Table S1. Single-phase and two-phase lipid extraction protocol for target sphingolipids. Table S2. Validation of optimized LC-MS/MS analysis for each analyte. LOD: limit of detection; LLOQ: limit of quantification; Intra: intra-day; Inter: inter-day. Table S3. Optimized MRM parameters for LC-MS/MS analysis. Table S4. Ultra performance liquid chromatography (UPLC) conditions for sphingolipid analysis. Table S5. Gene-specific primer sequences used for qPCR amplification. The primers were designed based on published gene sequences of *Homo sapiens* (NCBI Reference Sequence Database) using NCBI’s Primer-BLAST tool (https://www.ncbi.nlm.nih.gov/tools/primer-blast/, accessed on 3 September 2025). All primers were synthesized by Tsingke (Changsha, China) and validated via PCR a two-step quality control process. PCR amplification followed by Sanger sequencing to confirm target specificity, and gradient PCR optimization to determine optimal annealing temperatures. Primer efficiency was further evaluated using the standard curve method via qRT-PCR.

## Abbreviations

MA	myristic acid
SL	sphingolipid
Cer	ceramide
HexCer	hexosylceramide
SM	sphingomyelin
C14:0-CoA	myristoyl-CoA
GSL	glycosphingolipid
S1P	sphingoid base-1-phosphate
SPT	serine palmitoyltransferase
C16:0-CoA	palmitoyl-CoA
CERSs	ceramide synthases
DHCer	dihydroceramide
DEGS	desaturase enzyme
MRM	multiple reaction monitoring
GluCer	glucosylceramide
LacCer	lactosylceramide
LOD	limit of detection
LLOQ	lower limit of quantification
ACS	acyl-CoA synthetase
ELOVL	elongases of very long chain fatty acids
DHHexCer	dihydrohexosylceramide
DHLacCer	dihydrolactosylceramide
KDSR	ketosphinganine reductase
Sa1P	sphinganine-1-phosphate
UGCG	UDP-glucose ceramide glucosyltransferase
GBA	glucosylceramidase Beta
GLT	galactosyltransferase
SPHKs	sphingosine kinases
SGPP1	S1P phosphatase
SGPL1	S1P lyase 1
CERT	ceramide transporter
SMPD	sphingomyelin phosphodiesterase
SGMS	sphingomyelin synthase
