# Supplementary material for: Myristic Acid Remodels Sphingolipid Metabolism via Dual Pathways: Canonical d18-Sphingolipid Regulation and Non-Canonical d16-Sphingolipid Synthesis
Source: Nutrients. 2025 Sep 5;17(17):2881. doi: 10.3390/nu17172881 (PMC12429915; doi:10.3390/nu17172881)
Supplement: Supplementary file 1 [file nutrients-17-02881-s001.zip › nutrients-3805395-supplementary.pdf]

Figure S1

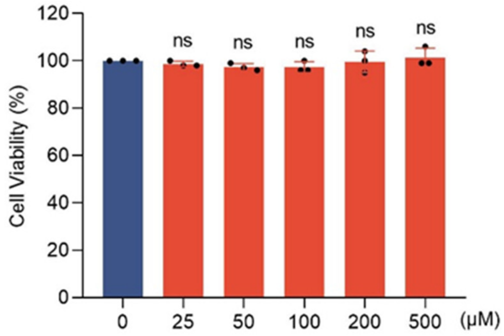

Figure S1. Cell viabilities of HepG2 cells treated with various concentration of myristic acid. The viability of HepG2 cells treated with myristic acid (MA) (0-500 μM, 24 h) was evaluated with the MTS assay, normalized to untreated controls. Statistical analysis via a two-tailed Student's *t*-test indicated no significant differences (all $p > 0.05$, ns) compared to the control. Data are from three independent biological replicate (black dots indicate individual replicates).

Figure S2

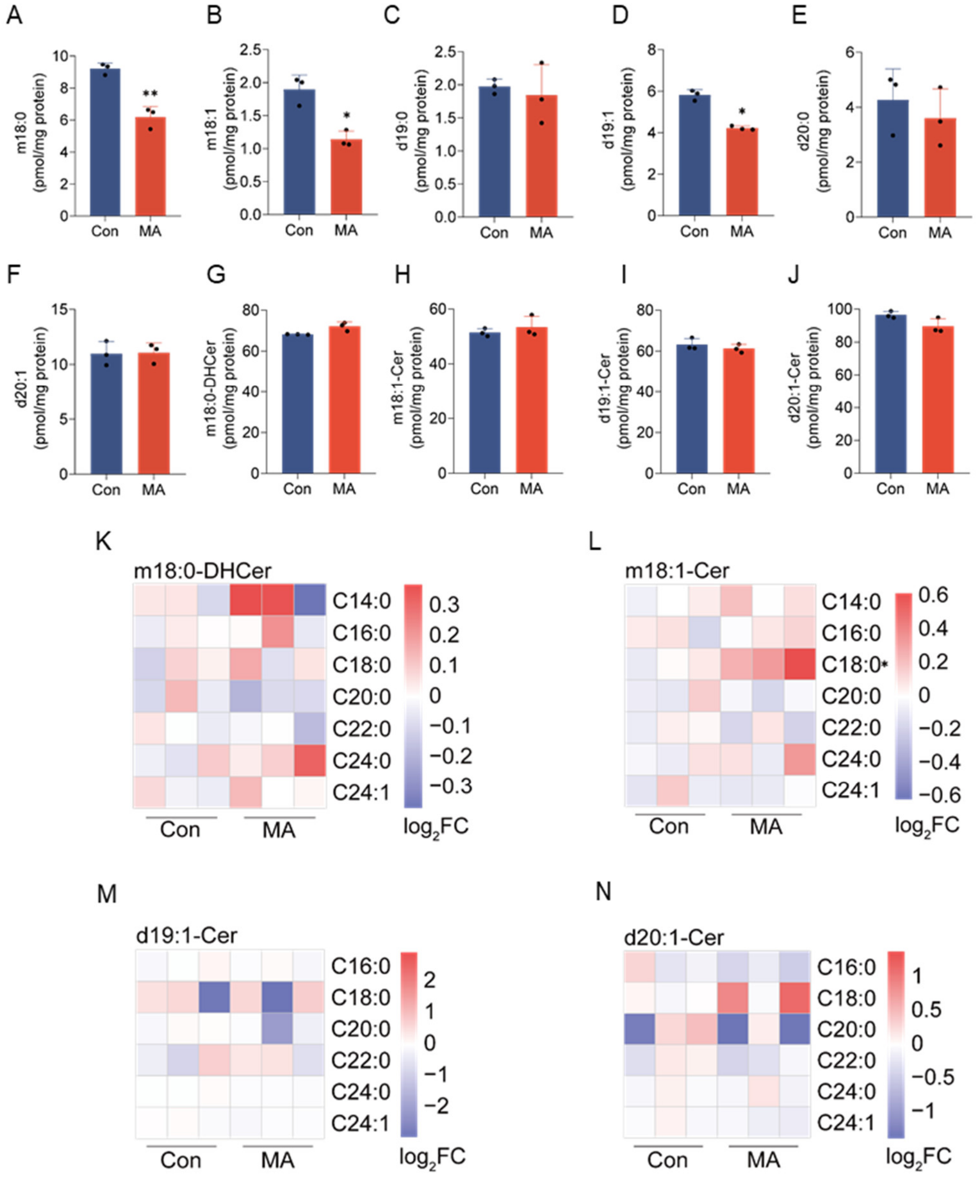

Figure S2. The effect of MA on non-canonical m18- 1-deoxysphingolipids and d19-, d20- sphingolipids. (A-B) Quantification of 1-deoxysphingoid bases showed reduced levels of (A) m18:0 and (B) m18:1 (units: pmol/mg protein). (C-F) Analysis of d19- and d20- sphingoid bases demonstrated a decrease in (D) d19:1, whereas (C) d19:0, (E) d20:0, and (F) d20:1 remained unchanged (units: pmol/mg protein). (G-J) Corresponding ceramide analysis showed that the levels of (G) m18:0-DHCer, (H) m18:1-Cer, (I) d19:1-Cer, and (J) d20:1-Cer were unaffected (units: pmol/mg protein). (K-L) Heatmaps of m18:0-DHCer (K) and m18:1-Cer (L) *N*-acyl chain distributions (C14~C24) as log_2-fold changes. (M-N) Heatmaps displaying d19:1-Cer (M) and d20:1-Cer (N) chain length modifications (C16~C24) as log_2-fold changes. For each panel, the maximum positive (red) log_2FC values and their corresponding fold changes are: 0.3 (~1.2-fold), 0.6 (~1.5-fold), 1 (2.0-fold), 2 (4.0-fold). A log_2FC value of 0 indicates no change from the mean, with positive and negative values representing upward and downward regulation, respectively. Color scale: all analyses were conducted in MA-treated HepG2 cells. Data are from three independent biological replicates (black dots indicate individual replicates). Significance was assessed by a two-tailed Student's *t*-test (* $p < 0.05$, ** $p < 0.01$).

Figure S3

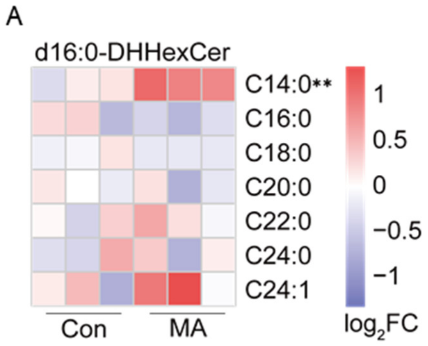

Figure S3. The effect of MA on d16:0-dihydrohexosylceramides (DHHexCer), and d16:0-dihydrolactosylceramides (DHLacCer). Heatmaps displaying (A) d16:0-DHHexCer and (B) d16:0-DHLacCer with C16~C24 acyl chain length as $\log_2$-fold changes. Color scale: all analyses were conducted in MA-treated HepG2 cells. Statistical significance was determined by two-tailed Student's *t*-test (n = 3 independent experiments), with * $p < 0.05$, and ** $p < 0.01$.

Figure S4

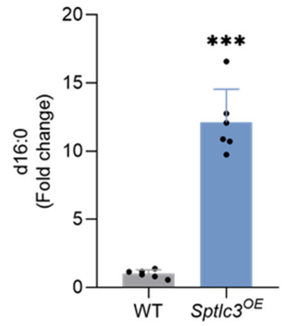

Figure S4. Fold change of sphinganine d16:0 in WT and *Sptlc3*^{OE} mice at 6 months of age. Liver samples were collected from wild type (WT) and *Sptlc3* over-expressed (*Sptlc3*^{OE}) C57BL/6J mice (SPF-grade, 6-month-old, males), with six mice per group (black dots indicate individual replicates). Statistical significance was determined by two-tailed Student's *t*-test, with *** $p < 0.001$.

Table S1. Single-phase and two-phase lipid extraction protocol for target sphingolipids

	Single-phase extraction	Two-phase extraction
Target sphingolipids	d16-/d18-/m18-/d19-/d20- sphingoid bases, and d16-/d18-phosphates	d16-/d18-Cers*, GSLs and SMs with chain lengths of C14:0, C16:0, C18:0, C20:0, C22:0, C24:0, and C24:1
Extraction solvents	1.5 mL Methanol:Dichloromethane (2:1, v/v)	1.5 mL Methanol:Chloroform (2:1, v/v)
Internal standard	10 μL Sphingolipid Mix II (Avanti Polar Lipids, #LM-6005, containing d17:0, d17:1, d17:0-Sa1P, d17:1-S1P, C12-Cer, C12-SM, C12-GluCer, and C12-LacCer)	
Incubation	48°C, overnight	
Saponification	150 μL 1 M KOH, 37°C shaking for 2 h	
Neutralization	Acetic acid ~8 μL	Acetic acid ~6 μL
Extraction	Centrifuge (1,000 rpm for 10 min)<br>Collect the supernatant	Add 2 mL Milli-Q water, Centrifuge (1,000 rpm for 10 min)<br>Collect the lower phase (chloroform phase)
Re-extraction (Twice)	1 mL Methanol:Dichloromethane (2:1, v/v) add to precipitate	1 mL Chloroform add to the remaining upper phase (water phase)

* Ceramides, glycosphingolipids (hexosylceramide and lactosylceramide), and sphingomyelins

Table S2. Validation of optimized LC-MS/MS analysis for each analyte

ID	Linear Range (nM) and linearity (R^2)	LOD (nM)	LLOQ (nM)	Assay Type (n = 3)	Recovery at different spike levels		CV (%)
					Spiked levels (n = 3) (nM)	Recoveries [Mean ± SD] %	
d17:1	5-1000 (0.9963)	0.08	3.86	Intra	100	98.91±3.27	3.31
				Inter	100	100.54±3.59	3.57
d17:0	5-1000 (0.9957)	0.09	4.40	Intra	100	100.19±3.10	3.09
				Inter	100	98.62±3.30	3.35
d17:1-S1P	5-1000 (0.9970)	0.10	5.24	Intra	100	99.58±3.20	3.21
				Inter	100	100.95±3.18	3.15
d17:0-Sa1P	5-1000 (0.9969)	0.09	4.59	Intra	100	98.58±2.40	2.43
				Inter	100	101.79±3.07	3.01
d18:1	5-1000 (0.9968)	0.07	3.66	Intra	100	98.69±2.83	2.86
				Inter	100	101.13±1.94	1.92
d18:0	5-1000 (0.9984)	0.10	5.09	Intra	100	99.56±4.52	4.54
				Inter	100	100.67±3.86	3.84
m18:0	5-1000 (0.9930)	0.07	3.27	Intra	100	99.20±1.57	1.59
				Inter	100	101.70±2.61	2.57
d18:1-C12:0 Cer	5-1000 (0.9906)	0.10	4.83	Intra	100	97.88±3.15	3.22
				Inter	100	100.33±2.62	2.61
d18:1-C12:0 SM	5-1000 (0.9919)	0.09	4.62	Intra	100	100.91±3.86	3.83
				Inter	100	99.50±3.22	3.24
d18:1-C12:0 HexCer	5-1000 (0.9922)	0.13	4.65	Intra	100	101.30±2.56	2.53
				Inter	100	98.17±2.18	2.22
d18:1-C12:0 LacCer	5-1000 (0.9921)	0.09	4.65	Intra	100	101.60±2.23	2.19
				Inter	100	99.44±4.40	4.42

LOD: limit of detection; LLOQ: limit of quantification; Intra: intra-day; Inter: inter-day

Table S3. Optimized MRM parameters for LC-MS/MS analysis

ID	Parent ion (*m/z*)	Fragment ion (*m/z*)	Declustering Potential (V)	Collision Energy (V)
d16:0	274.3	256.3	105	20
d16:1	272.3	254.3	70	15
d16:0-C14:0 DHCer	484.5	238.3	85	40
d16:0-C16:0 DHCer	512.5	238.3	90	35
d16:0-C18:0 DHCer	540.5	238.3	85	30
d16:0-C20:0 DHCer	568.6	238.3	80	45
d16:0-C22:0 DHCer	596.6	238.3	80	45
d16:0-C24:0 DHCer	624.6	238.3	115	35
d16:0-C24:1 DHCer	622.6	238.3	115	35
d16:1-C14:0 Cer	482.5	236.3	80	30
d16:1-C16:0 Cer	510.5	236.3	90	30
d16:1-C18:0 Cer	538.5	236.3	85	30
d16:1-C20:0 Cer	566.6	236.3	115	45
d16:1-C22:0 Cer	594.6	236.3	120	45
d16:1-C24:0 Cer	622.6	236.3	115	35
d16:1-C24:1 Cer	620.6	236.3	80	50
d17:0 Sa1P	368.3	270.3	100	20
d17:1 S1P	366.3	250.3	100	35
d16:0 Sa1P	354.3	256.3	75	20
d16:1 S1P	352.3	236.3	80	20
d16:1-C14:0 HexCer	644.5	236.3	100	50
d16:1-C16:0 HexCer	672.5	236.3	100	50
d16:1-C18:0 HexCer	700.6	236.3	80	80
d16:1-C20:0 HexCer	728.6	236.3	95	50
d16:1-C22:0 HexCer	756.6	236.3	95	50
d16:1-C24:0 HexCer	784.7	236.3	105	50
d16:1-C24:1 HexCer	782.7	236.3	80	55
d16:1-C14:0 LacCer	806.6	236.3	100	50
d16:1-C16:0 LacCer	834.6	236.3	85	55
d16:1-C18:0 LacCer	862.7	236.3	90	60
d16:1-C20:0 LacCer	890.7	236.3	100	55
d16:1-C22:0 LacCer	918.7	236.3	100	50
d16:1-C24:0 LacCer	946.8	236.3	105	50
d16:1-C24:1 LacCer	944.8	236.3	80	55
d16:0-C14:0 DHHexCer	646.5	238.3	100	50
d16:0-C16:0 DHHexCer	674.5	238.3	100	50
d16:0-C18:0 DHHexCer	702.6	238.3	80	50
d16:0-C20:0 DHHexCer	730.6	238.3	95	50
d16:0-C22:0 DHHexCer	758.6	238.3	95	50
d16:0-C24:0 DHHexCer	786.7	238.3	105	50

ID	Parent ion (*m*/*z*)	Fragment ion (*m*/*z*)	Declustering Potential (V)	Collision Energy (V)
d16:0-C24:1 DHHexCer	784.7	238.3	80	55
d16:0-C14:0 DHLacCer	808.6	238.3	100	50
d16:0-C16:0 DHLacCer	836.6	238.3	100	50
d16:0-C18:0 DHLacCer	864.7	238.3	80	50
d16:0-C20:0 DHLacCer	892.7	238.3	95	50
d16:0-C24:0 DHLacCer	948.8	238.3	105	50
d16:0-C24:1 DHLacCer	946.8	238.3	80	55
d17:0	288.3	270.3	100	15
d17:1	286.3	268.3	110	15
d18:0	302.3	284.3	90	25
d18:1	300.3	282.3	100	15
d18:0-C14:0 DHCer	512.5	266.3	100	35
d18:0-C16:0 DHCer	540.5	266.3	100	35
d18:0-C18:0 DHCer	568.6	266.3	100	35
d18:0-C20:0 DHCer	596.6	266.3	100	35
d18:0-C22:0 DHCer	624.6	266.3	100	35
d18:0-C24:0 DHCer	652.7	266.3	100	35
d18:0-C24:1 DHCer	650.6	266.3	100	35
d18:1-C12:0 Cer	482.5	264.3	100	35
d18:1-C14:0 Cer	510.5	264.3	100	35
d18:1-C16:0 Cer	538.5	264.3	100	35
d18:1-C18:0 Cer	566.6	264.3	100	35
d18:1-C20:0 Cer	594.6	264.3	100	35
d18:1-C22:0 Cer	622.6	264.3	100	35
d18:1-C24:0 Cer	650.6	264.3	100	35
d18:1-C24:1 Cer	648.6	264.3	100	35
d18:0 S1P	382.3	284.3	95	10
d18:1 S1P	380.3	264.3	80	20
d18:0-C14:0 DHHexCer	674.5	266.3	70	40
d18:0-C16:0 DHHexCer	702.6	266.3	65	45
d18:0-C18:0 DHHexCer	730.6	266.3	60	45
d18:0-C20:0 DHHexCer	758.7	266.3	70	50
d18:0-C22:0 DHHexCer	786.7	266.3	70	50
d18:0-C24:0 DHHexCer	814.8	266.3	110	60
d18:0-C24:1 DHHexCer	812.7	266.3	70	50
d18:0-C14:0 DHLacCer	836.6	266.3	60	60
d18:0-C16:0 DHLacCer	864.6	266.3	60	60
d18:0-C18:0 DHLacCer	892.7	266.3	75	55
d18:0-C20:0 DHLacCer	920.7	266.3	90	65
d18:0-C22:0 DHLacCer	948.7	266.3	70	55
d18:0-C24:0 DHLacCer	976.8	266.3	75	65
d18:0-C24:1 DHLacCer	974.8	266.3	75	55

ID	Parent ion (m/z)	Fragment ion (m/z)	Declustering Potential (V)	Collision Energy (V)
d18:1-C14:0 HexCer	672.5	264.3	70	40
d18:1-C16:0 HexCer	700.6	264.3	65	45
d18:1-C18:0 HexCer	728.6	264.3	60	45
d18:1-C20:0 HexCer	756.7	264.3	70	50
d18:1-C22:0 HexCer	784.7	264.3	70	50
d18:1-C24:0 HexCer	812.8	264.3	110	60
d18:1-C24:1 HexCer	810.7	264.3	70	50
d18:1-C12:0 LacCer	806.6	264.3	77	49
d18:1-C14:0 LacCer	834.6	264.3	85	45
d18:1-C16:0 LacCer	862.6	264.3	70	55
d18:1-C18:0 LacCer	890.7	264.3	85	55
d18:1-C20:0 LacCer	918.7	264.3	70	65
d18:1-C22:0 LacCer	946.7	264.3	70	60
d18:1-C24:0 LacCer	974.8	264.3	60	55
d18:1-C24:1 LacCer	972.7	264.3	60	55
d30:0-SM	649.5	184.3	115	35
d30:1-SM	647.5	184.3	115	35
d30:2-SM	645.5	184.1	115	35
d32:0-SM	677.6	184.3	100	35
d32:1-SM	675.5	184.3	100	35
d32:2-SM	673.5	184.3	100	35
d34:0-SM	705.6	184.3	120	35
d34:1-SM	703.6	184.3	120	35
d34:2-SM	701.6	184.3	120	35
d36:0-SM	733.6	184.3	90	35
d36:1-SM	731.6	184.3	90	35
d36:2-SM	729.6	184.3	90	35
d38:0-SM	761.7	184.3	80	35
d38:1-SM	759.6	184.3	80	35
d38:2-SM	757.6	184.3	80	35
d40:0-SM	789.7	184.3	100	40
d40:1-SM	787.7	184.3	100	40
d40:2-SM	785.7	184.3	100	40
d42:0-SM	817.7	184.3	85	55
d42:1-SM	815.7	184.3	85	55
d42:2-SM	813.7	184.3	85	55
d44:0-SM	845.7	184.3	85	45
d44:1-SM	843.7	184.3	85	45
d44:2-SM	841.7	184.3	85	45
m18:0	286.3	268.3	70	25
m18:1	284.3	266.3	115	15
d19:0	316.3	298.3	70	15

ID	Parent ion (m/z)	Fragment ion (m/z)	Declustering Potential (V)	Collision Energy (V)
d20:0	330.3	312.3	85	20
d20:1	328.3	310.3	115	30
m18:0-C14:0 DHCer	496.5	268.3	120	50
m18:0-C16:0 DHCer	524.5	268.3	80	35
m18:0-C18:0 DHCer	552.6	268.3	80	30
m18:0-C20:0 DHCer	580.6	268.3	85	45
m18:0-C22:0 DHCer	608.6	268.3	80	35
m18:0-C24:0 DHCer	636.7	268.3	120	40
m18:0-C24:1 DHCer	634.7	268.3	85	35
m18:1-C14:0 Cer	494.5	266.3	110	35
m18:1-C16:0 Cer	522.5	266.3	120	40
m18:1-C18:0 Cer	550.6	266.3	125	40
m18:1-C20:0 Cer	578.6	266.3	125	35
m18:1-C22:0 Cer	606.6	266.3	105	40
m18:1-C24:0 Cer	634.7	266.3	80	55
m18:1-C24:1 Cer	632.7	266.3	110	35
d19:1-C16:0 Cer	552.5	278.3	115	50
d19:1-C18:0 Cer	580.6	278.3	95	45
d19:1-C20:0 Cer	608.6	278.3	110	55
d19:1-C22:0 Cer	636.6	278.3	95	40
d19:1-C24:0 Cer	664.6	278.3	95	40
d19:1-C24:1 Cer	662.6	278.3	95	45
d20:1-C16:0 Cer	566.5	292.3	80	40
d20:1-C18:0 Cer	594.6	292.3	100	55
d20:1-C20:0 Cer	622.6	292.3	100	35
d20:1-C22:0 Cer	650.6	292.3	85	40
d20:1-C24:0 Cer	678.6	292.3	90	45
d20:1-C24:1 Cer	676.6	292.3	100	40

Table S4. Ultra performance liquid chromatography (UPLC) conditions for sphingolipid analysis

	Single-phase extraction			Two-phase extraction		
Target sphingolipids	d16-/d18-/m18-/d19-/d20- sphingoid bases, and d16-/d18-phosphates			d16-/d18-Cers*, GSLs and SMs with chain lengths of C14:0, C16:0, C18:0, C20:0, C22:0, C24:0, and C24:1		
Column	C18 column (2.6 µm, 100 × 1.7 mm, 100Å, Phenomenex)					
Mobile phase A	Methanol:Milli-Q water:Formic acid = 58:41:1 (v/v/v)<br>5 mM ammonium formate			Methanol:Milli-Q water:Acetonitrile = 1:1:1 (v/v/v)<br>7 mM ammonium acetate		
Mobile phase B	Methanol:Formic acid = 99:1 (v/v)<br>5 mM ammonium formate			Isopropanol<br>7 mM ammonium acetate		
Pre-equilibration	50% mobile phase B, 2 min					
Gradient program#	25%	0.0-1.0	min	50%	0.0-1.0	min
	25%-100%	1.0-3.0	min	50%-98%	1.0-8.0	min
	100%	3.0-5.0	min	98%	8.0-11.0	min
	100%-25%	5.0-5.8	min	98%-50%	11.0-11.1	min
	25%	5.8-6.0	min	50%	11.1-12.0	min
Flow rate	0.5 mL/min					
Injection volume	2 µL					

* Ceramides, glycosphingolipids (hexosylceramide and lactosylceramide)), and sphingomyelins

Mobile phase B for gradient elution

Table S5. Gene-specific primer sequences used for qPCR amplification

Gene	Primers (5′→3′)
SPTLC1	F: GCAGTGTTGAAGGAAAAGTGCGG<br>R: CAGTGCTCTCTTCCAGTTGTAGG
SPTLC2	F: CCAGACTGTCAGGAGCAACCAT<br>R: TTCGTGTCCGAGGCTGACCATA
SPTLC3	F: CCGACTCTCAGGTGCAACCATA<br>R: TGTAGACACCCTCCACCAGGAT
ELOVL1	F: GTCTACAACTTCTCACTGGTGGC<br>R: AAGTGCCTCAGGGCTGTTGGAA
ELOVL2	F: CAGTGTCAAGATCTTACCAGCG<br>R: CCAGGAACTCTACTGATTTGGAG
ELOVL3	F: CTACCTGGTTCTCATCGCTGTG<br>R: GTAGCACAGTCCCCATAATGCC
ELOVL5	F: ACGTCTACCACCATGCCTCGAT<br>R: TGGAAAGGGACTGACGACAAACC
ELOVL6	F: CCATCCAATGGATGCAGGAAAAC<br>R: CCAGAGCACTAATGGCTTCCTC
ELOVL7	F: CCTACTATGGACTTTCTGCATTGG<br>R: GAACTGGCTTATGTGGATGGCG
DEGS1	F: CCTGGGTTTGCACCCAATTT<br>R: GAAACTCCAGCTCAGTGGAAGA
DEGS2	F: GATCTCGAGGTGGCGTCTTAC<br>R: ACCGACAGGGTCATTCTCCC
CERS1	F: GTCACCCTGCAACCGTGCCAC<br>R: AGGTCGAAGACGACTGTCCACT
CERS2	F: GCCTTGCTCTTCCTCATCGTTC<br>R: TGCTTGCCACTGGTCAGGTAGA
CERS4	F: GACCTTCTCCTACAGTGCCAAC<br>R: GTCGCACACTTGCTGATACTGC
CERS5	F: GGTCACCATTGGGCTTATCTCC<br>R: GTGTCACAGAGCCGCTGATACT
CERS6	F: GACGCAATCAGGAGAAGCCAAG<br>R: GGTAGTTGTACCAGCAATGCCTC
SPHK1	F: CCAAGTGTTGGGAAACGGG<br>R: AGTCGAGAAGAGGCAGGTCC
SPHK2	F: TTATCAGGTTCACCGCTCCC<br>R: GCTGGTAGGAGCAAGGAGTC
SGPP1	F: CATCTGGGTGCTGGTCATGT<br>R: GGAGGGCATGCTGTACTCAG
SGPL1	F: CTCGAGGGAAGGAGACTGGA<br>R: CATGCAATTAGCTGCCAGGG
CERT	F: AGTATTAACAGCTCAGAGGATGAA

Gene	Primers (5′→3′)
	R: GCAGTGAATCCAAAGCTCGC
SGMS1	F: CGAAAGCGTTCGCACCAG
	R: GCTGTCGTCACGTTGCAC
SGMS2	F: GTGTGCTCCAAAGCTCAATGG
	R: GTGTGACCGCTGAAGAGGAA
SMPD1	F: GCTGGCTCTATGAAGCGATGGC
	R: AGAGCCAGAAGTTCTCACGGGA
SMPD2	F: GGGACGAGCTTGGAGGAAAA
	R: CCCCAGACCGTTCCTTCTTC
SMPD3	F: GCTGAGTCTGAGGGAGGCT
	R: GAAGAAGAGCTGTCACCGCA
GAPDH	F: AGGTCGGTGTGAACGGATTTG
	R: TGTAGACCATGTAGTTGAGGTCA
